# Transepithelial Transport of Curcumin in Caco-2 Cells Is significantly Enhanced by Micellar Solubilisation

**DOI:** 10.1007/s11130-016-0587-9

**Published:** 2016-11-29

**Authors:** Jan Frank, Christina Schiborr, Alexa Kocher, Jürgen Meins, Dariush Behnam, Manfred Schubert-Zsilavecz, Mona Abdel-Tawab

**Affiliations:** 1 Institute of Biological Chemistry and Nutrition, University of Hohenheim, D-70599 Stuttgart, Germany; 2 Central Laboratory of German Pharmacists, Carl-Mannich-Str. 20, D-65760 Eschborn, Germany; 3 AQUANOVA AG, D-64295 Darmstadt, Germany; 4 Institute of Pharmaceutical Chemistry, University of Frankfurt, D-60438 Frankfurt, Germany

**Keywords:** Absorption, Bioavailability, Caco-2 cells, Curcumin micelles, Transepithelial transport, Tween 80

## Abstract

Curcumin, the active constituent of *Curcuma longa* L. (family Zingiberaceae), has gained increasing interest because of its anti-cancer, anti-inflammatory, anti-diabetic, and anti-rheumatic properties associated with good tolerability and safety up to very high doses of 12 g. Nanoscaled micellar formulations on the base of Tween 80 represent a promising strategy to overcome its low oral bioavailability. We therefore aimed to investigate the uptake and transepithelial transport of native curcumin (CUR) vs. a nanoscaled micellar formulation (Sol-CUR) in a Caco-2 cell model. Sol-CUR afforded a higher flux than CUR (39.23 vs. 4.98 μg min^−1^ cm^−2^, respectively). This resulted in a higher P_app_ value of 2.11 × 10^−6^ cm/s for Sol-CUR compared to a P_app_ value of 0.56 × 10^−6^ cm/s for CUR. Accordingly a nearly 9.5 fold higher amount of curcumin was detected on the basolateral side at the end of the transport experiments after 180 min with Sol-CUR compared to CUR. The determined 3.8-fold improvement in the permeability of curcumin is in agreement with an up to 185-fold increase in the AUC of curcumin observed in humans following the oral administration of the nanoscaled micellar formulation compared to native curcumin. The present study demonstrates that the enhanced oral bioavailability of micellar curcumin formulations is likely a result of enhanced absorption into and increased transport through small intestinal epithelial cells.

## Introduction

Curcumin, the active constituent of *Curcuma longa* L. (family Zingiberaceae), has gained increasing interest because of its anti-inflammatory, anti-diabetic, anti-rheumatic, anti-oxidant, anti-cancer, wound healing, and hepatoprotective properties associated with good tolerability and safety up to very high doses of 12 g [1–4].

However, its low aqueous solubility, extensive intestinal and hepatic metabolism, as well as rapid urinary excretion limit its oral bioavailability to plasma concentrations in the nanomolar range, even when ingested at single oral doses of 10–12 g [5, 6].

In order to overcome the low bioavailability of curcumin, a number of different strategies have been pursued to enhance its solubility, including the use of adjuvants to inhibit its metabolism [7], the application of crystalline curcumin in micronized form [8] and the incorporation of curcumin into phosphatidylcholine liposomes [9] or micelles [10, 11]. Nanoencapsulation of curcumin, using synthetic and natural-based polymers has been mainly used for intravenous cancer therapy [12]. Only two studies reported improved bioavailability of PLGA-PEG encapsulated curcumin nanoparticles and a liposomal nanoparticle formulation in rats following oral administration [13, 14]. Recently, curcumin-loaded self-assembled polymeric micelles were prepared using di-tocopherol polyethylene glycol 2000 succinate (TPGS), HS15 (octadecanoic acid, 12-hydroxy-polymer with alpha-hydro-omega-hydroxypoly (oxy-1,2-ethanediyl)) and Pluronic F127 [15]. Another promising approach for the efficient delivery of poorly soluble substances is the preparation of nanoscaled micellar formulations based on Tween 80, which enhance curcumin bioavailability up to 185-fold in humans [10, 11]. We therefore investigated the transepithelial transport and cellular accumulation of curcumin, demethoxycurcumin (DMC, curcumin II) and *bis-*demethoxycurcumin (BDMC, curcumin III) in their native form compared to a nanoscaled micellar formulation in a Caco-2 cell model.

## Materials and Methods

### Curcumin Formulations

Native curcumin powder composed of 82 % curcumin, 16 % DMC and 2 % BMDC used for the transport studies and for the preparation of the Tween 80 based micellar formulation was purchased from Jupiter Lys (Cochin, Kerala State, India). Sol-CUR consisting of 7 % curcuminoid powder (equal to 6 % curcumin) and 93 % Tween-80 (Kolb, Hedingen, Switzerland) was produced by AQUANOVA AG (Darmstadt, Germany). A dose of 98 mg total curcuminoids (80.36 mg curcumin, 15.68 mg DMC and 1.96 mg BDMC) was used for the transport experiments with native and micellar curcumin.

The sample solution of Sol-CUR used for the transport experiments was prepared by diluting 53 mg of Sol-CUR with 10 mL modified FaSSIF (fasted state simulated intestinal fluid) transport buffer. The sample solution of CUR was prepared by diluting 32 mg native curcumin powder with 10 mL modified FaSSIF transport buffer and further diluting an aliquot of 1 mL from that solution with 9 mL modified FaSSIF transport buffer. Both sample solutions yielded a curcumin concentration of 320 μg/mL (869 μmol/L) corresponding to a dose of 80 mg curcumin taken together with a glass of 250 mL water.

### Cell Culture Conditions

Differentiated Caco-2 cells (passages 24–36; American Type Culture Collection (ATCC), Maryland, USA) were plated at a density of 6.5 × 10^4^ cells per cm^2^ on 12-well Transwell plates (1.2 cm^2^ polycarbonate membrane, 0.4 μm pore size; Corning, NY, USA) and grown in Dulbecco’s modified Eagle’s medium (DMEM) containing 25 mM glucose supplemented with 10 % FCS (fetal calf serum), 1 % NEAA (non essential amino acids) and 0.01 % gentamycin (all from Biochrom AG, Berlin, Germany) in an atmosphere consisting of 90 % relative humidity with 10 % CO_2_ at 37 °C.

Modified FaSSIF (fasted state simulated intestinal fluid), containing 3 mM taurocholate sodium and 0.75 mM phosphatidylcholine in dihydrogenphosphate sodium buffer (28.66 mM), was prepared by diluting appropriate amounts of Phares SIF powder (Phares AG, Muttenz, Switzerland) with HBSS transport medium, composed of Hanks balanced salt solution (HBSS; Biochrom AG, Berlin, Germany) containing 20 mM HEPES (4-(2-hydroxyethyl)-1-piperazineethanesulfonic acid; Sigma-Aldrich, Steinheim, Germany) buffered at a pH of 7.4.

### Permeability Experiments

Permeation was investigated in apical-to-basolateral direction (AB) at 37 °C (*n* = 6 each). During transport the plates were agitated on a shaker at 120 rpm and kept at a constant temperature of 37 °C. Prior to each experiment, the Caco-2 monolayers were washed with HBSS. The transepithelial electrical resistance (TEER) was measured before and after the transport experiments. FITC–Dextran (mean MW 4400 g/mol, Sigma-Aldrich) was used to gauge the integrity of the monolayers and propranolol hydrochloride (Fagron, Barsbüttel, Germany) served as positive control for the functionality of every cell passage (*n* = 6). The validity of the Caco-2 system in the present study was ensured by TEER values greater 250 Ω cm^2^ and an average P_app_ value for propranolol of 56.77 × 10^−6^ cm/s [16].

To estimate the mass flux, the receiver fluid (1500 μL) was withdrawn after 15, 30, 45, 60, 90, 120 and 180 min, and replaced by an equal volume of fresh buffer solution, respectively. The donor fluid (500 μL) was removed at the end of the transport experiments to determine the remaining apical curcuminoid concentrations. Furthermore each transwell filter was vortexed in 0.5 mL methanol for 15 min at the end of the incubation time. The resulting cell lysate was subjected to HPLC analysis to determine the accumulated curcuminoids in the Caco-2 cells. In addition, the initial curcuminoid concentrations (t_0_) were determined in the CUR and Sol-CUR sample solution prior to the transport experiments.

### Quantification of Curcumin

Sample preparation and analysis of curcuminoids were previously reported in detail [10, 11]. Briefly, one mL fluid was acidified with 10 μL 6 M hydrochloric acid and incubated with 100 μL beta-glucuronidase type H1 from *Helix pomatia* (1 mg/100 μL in 0.1 M sodium acetate buffer, Sigma-Aldrich Chemie GmbH, Schnelldorf, Germany) for 45 min at 37 °C. After triplicate extraction with 95 % ethyl acetate and 5 % methanol (*v*/v), supernatants were evaporated to dryness and resuspended in 150 μL methanol, vortexed for 20 s, stored in the dark for 10 min, vortexed for 20 s and transferred to HPLC vials. Twenty μL of each sample was injected into the HPLC system.

Curcuminoids were quantified on a Jasco HPLC system (Jasco GmbH, Gross-Umstadt, Germany) with a fluorescence detector (excitation wavelength 426 nm, emission wavelength 536 nm) and separated on a Reprosil-Pur C18-AQ column (150 mm × 4 mm, 3 μm particle size; Dr. Maisch GmbH, Ammerbuch, Germany) maintained at 40 °C. The mobile phase consisted of 52 % de-ionized water (adjusted to pH 3 with perchloric acid), 34 % acetonitrile and 14 % methanol. Curcuminoids were quantified against external standard curves (curcumin, purity ≥97.2 %, CAS # 458–37-7; DMC, purity ≥98.3 %, CAS # 22,608–11-13; BDMC, purity ≥99.4 %, CAS # 24,949–16-; Chromadex, Irvine, USA).

### Calculation of the Flux and Permeability Coefficient

The flux was calculated over the linear range of the permeation curve for each well using the formula (dc/dt) × (1/A), where dc/dt indicates the slope of the permeation and A the surface area of the monolayer.

The permeability coefficient P_app_ (cm/s) was calculated as follows: (dC/dt) × (Vr/AC0), where dc/dt is the flux rate (μg/(mL × s)) through the monolayer, Vr is the volume of the receiver chamber (mL), A is the surface area of the cell monolayer, and C_0_ is the initial concentration of the donor fluid (μg/mL).

## Results and Discussion

**Fig. 1:**
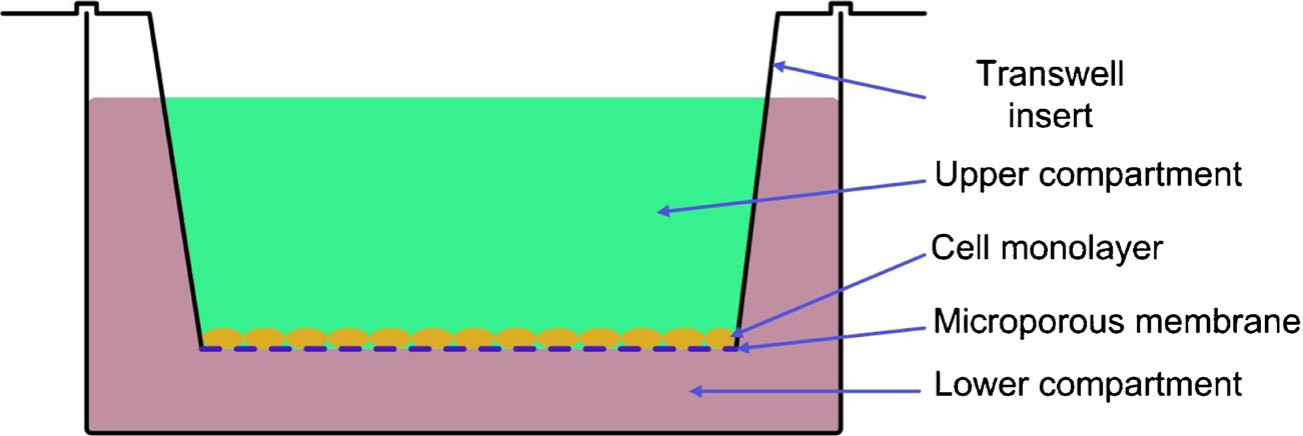
Experimental setting of the Caco-2 transport experiments

The Caco-2 cell system, being recommended by the US Food and Drug Administration (FDA), is an established and widely accepted *in vitro* tool for predicting the intestinal absorption of organic substances (Fig. 1) [17–19]. In the present study, curcumin was dissolved in modified FASSIF to simulate the gastrointestinal content at the site of absorption in the fasted state. No additional solubility enhancing surfactants were used as modified FASSIF already contains taurocholate sodium and lecithin as solubilizing enhancers.

**Fig. 2:**
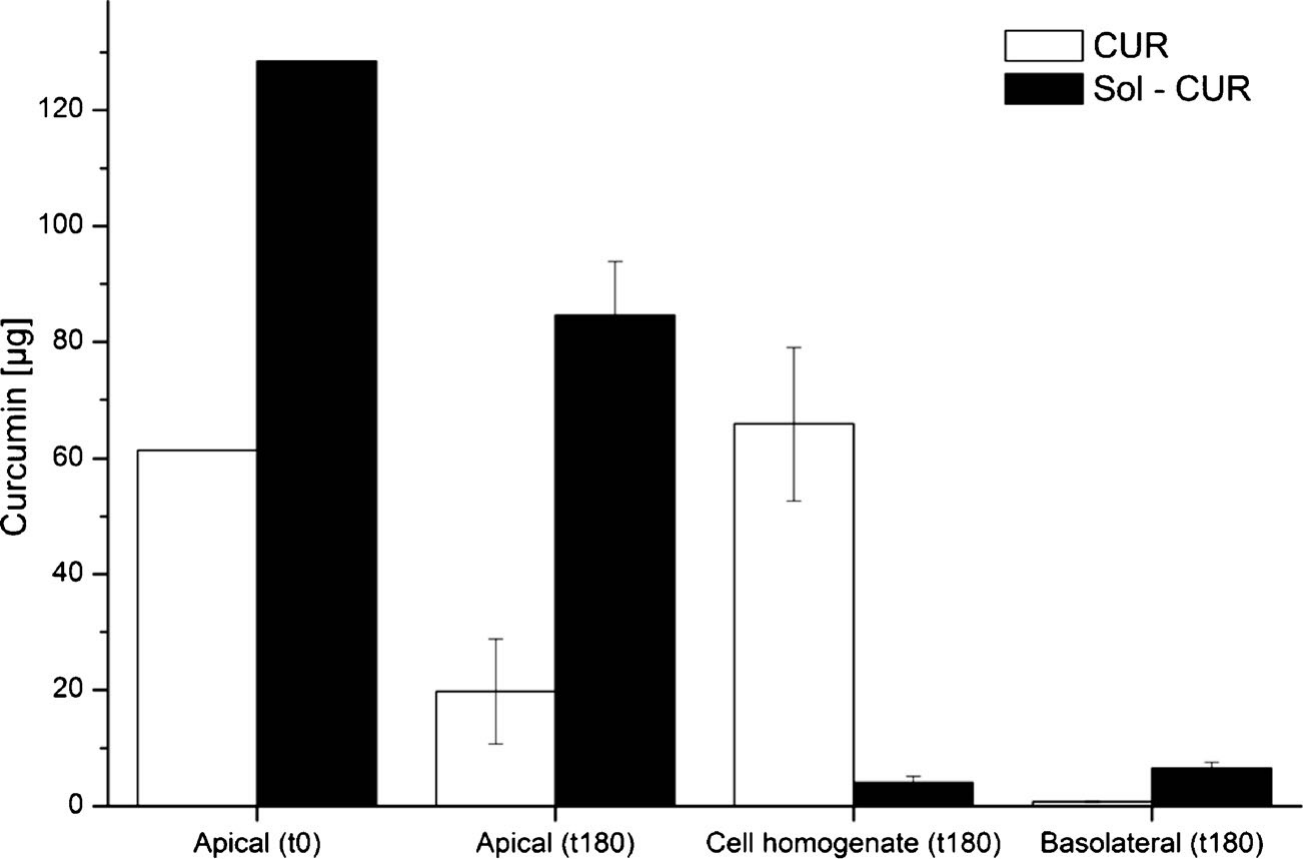
Curcumin concentrations (*n* = 6; mean ± SD) in the apical and basolateral compartments and in the cell pellets of Caco-2 cells incubated for 180 min with identical doses of native (CUR) and nanoscaled micellar curcumin (Sol-CUR)

At the end of the transport experiments, Sol-CUR afforded a nearly 9.5 fold higher concentration of curcumin on the basolateral side (transepithelial transport) compared to CUR, while the amount of curcumin accumulated in the Caco-2 cells was much lower with Sol-CUR than CUR (Fig. 2), suggesting that the nanoscaled micellar formulation does not only increase the cellular uptake, but also the basolateral secretion of curcumin.

**Fig. 3:**
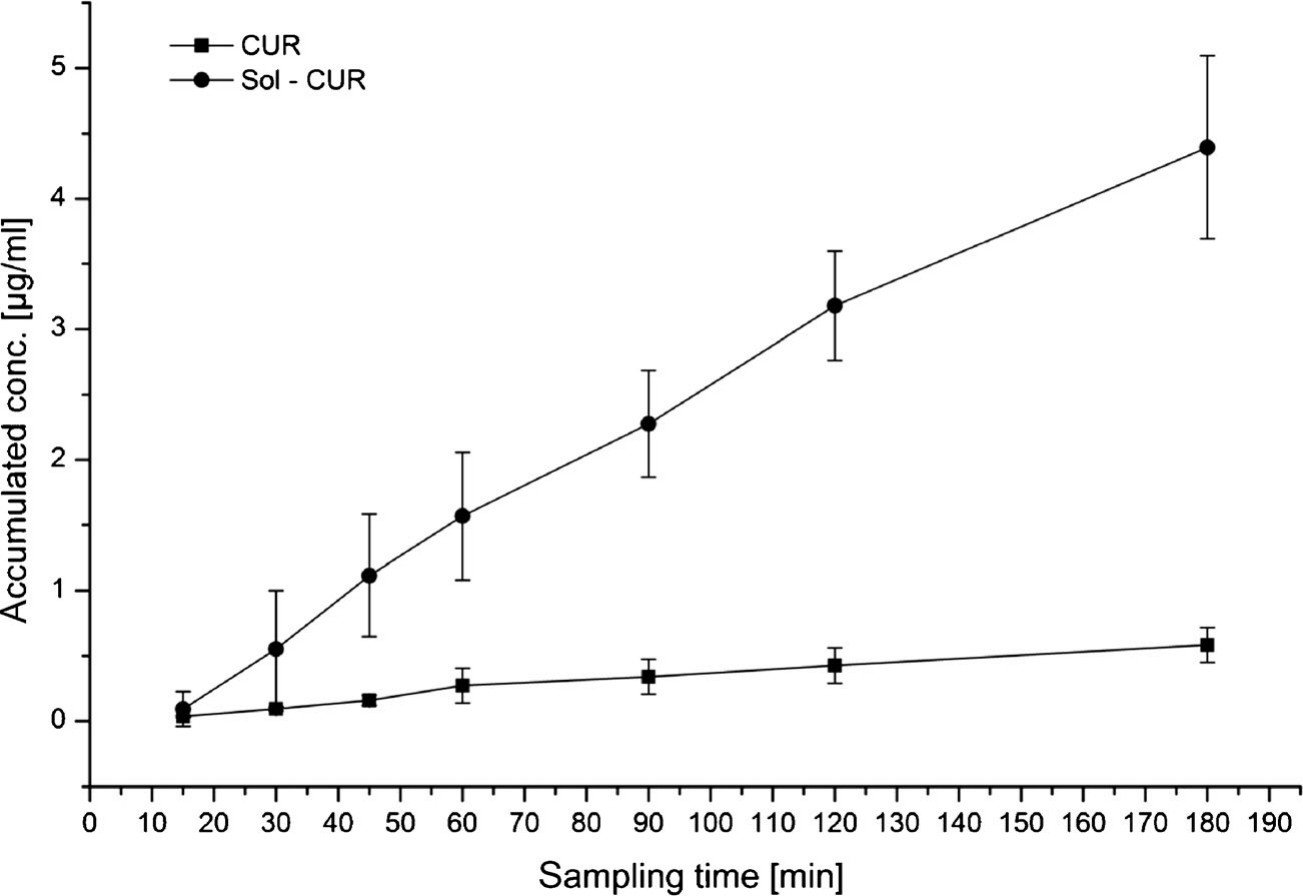
Curcumin flux (μg min^−1^ cm^−2^) of native (CUR) and nanoscaled micellar curcumin. Sol-CUR across Caco-2 monolayers (*n* = 6; mean ± S.D.)

The higher amount of Sol-CUR on the basolateral site results from a higher transport rate compared to CUR and a higher flux of 39.23 μg min^−1^ cm^−2^ for Sol-CUR compared to 4.98 μg min^−1^ cm^−2^ for CUR (Fig. 3). P_app_ values for Sol-CUR and CUR were 2.11 × 10^−6^ cm/s and 0.56 × 10^−6^ cm/s, respectively. Also, the permeability of Caco-2 cells for BDMC and DMC was increased as demonstrated by the higher P_app_ values obtained for BDMC and DMC in the nanoscaled micellar formulation compared to native curcumin (Table 1).

**Table 1:** P_app_ values (expressed as 10^−6^ cm/s) calculated for curcumin, DMC and BDMC in native curcumin (CUR) and nanoscaled micellar formulation of curcumin (Sol-CUR)

Analyte	CUR	Sol-CUR
Curcumin	0.56 ± 0.14	2.11 ± 0.34
DMC	1.32 ± 0.11	3.61 ± 0.59
BDMC	1.91 ± 0.25	6.63 ± 0.67

Compared to the P_app_ values for native curcumin of 0.05 × 10^−6^ cm/s and 0.07 × 10^−6^ cm/s reported in the literature by Wang et al. and Dempe et al., respectively, the P_app_ value determined in our study was higher [15, 20]. These differences may be attributed to differences in the experimental conditions. Transport experiments in both studies were performed with HBSS on the apical side, which is supplemented with glucose (25 mM) and buffered with 10 mM HEPES at a pH of 7.4. Although frequently used in Caco-2 transport experiments, this buffer does not reflect the physiological conditions *in vivo*, since the absence of bile salts may limit the solubility of highly lipophilic substances and may thus affect the outcome of the Caco-2 transport experiments [21]. Moreover this buffer promotes adsorption and/or non-specific binding of highly lipophilic substances to device surfaces, especially in the absence of sink conditions [22]. In order to overcome these drawbacks, the Caco-2 model used in this study was adapted to more closely mimic physiological conditions. This implied the provision of sink conditions as *in vivo*, *i.e*., a sufficiently diluted dissolution system in the receiver compartment that does not impede the dissolution of curcumin by approaching saturation. The addition of 4% BSA provided the necessary driving force, similar to *in vivo*, where the drug absorbed across the intestinal epithelium is immediately carried away by the portal blood containing about 4% albumin [21]. Further adaptation to *in vivo* conditions was achieved by using modified FASSIF instead of pure HBSS in the apical compartment. The taurocholate sodium and lecithin included in modified FASSIF simulate the colloidal mixture of bile salts and phosphatidylcholine in the intestine, which are responsible for enhanced solubilisation effects *in vivo*. Thus, the enhanced initial solubility of curcumin in the apical compartment in combination with the provided sink conditions, led to a higher P_app_ value for native curcumin than that published before [15, 20]. A higher P_app_ value for native curcumin of 2.9 × 10^−6^ cm/s was reported only in one study, which may be explained by the much higher initial concentration of curcumin of 170 μmol/L used, a transition time of only 120 min and a pH of 6.5 in the apical compartment at which curcumin is more stable [23].

The P_app_ value of 2.11 × 10^−6^ cm/s for Sol-CUR observed in the present study, indicating a 3.8-fold improvement in the permeability of micellar curcumin over the native form, suggests that the enhanced oral bioavailability of micellar curcumin formulations reported in two human pharmacokinetic studies, resulting in an up to 185-fold increase in the AUC compared to native curcumin [10, 11], is likely a result of enhanced absorption into and increased transport through small intestinal epithelial cells. The present study is the first to provide insights into the processes underlying the observed enhanced bioavailability of nanoscaled micellar curcumin formulations in humans, underlining thus the potential of the Caco-2 cell model to serve as an excellent predictive tool for the oral absorption of not only native compounds, but also more complex drug-excipient formulations.

Also curcumin-loaded self-assembled polymeric micelles made from TPGS2 K, HS15, and Pluronic F127 facilitated a 3.5- fold increase in permeability compared to native curcumin [15]. In fact, both TPGS2 K and Tween 80 are non-ionic surfactants improving the bioavailability of poorly soluble drugs by micellar solubilisation [23]. However, Tween 80-based micellar formulations are characterized by a better surfactant to curcumin ratio. While the curcumin-loaded self-assembled polymeric micelles consisted of curcumin, TPGS2 K, HS15 and Pluronic F127 in a ratio of 1:10:10:2 (ca. 4% curcuminoids corresponding to 3% curcumin) [15], the Tween 80 micellar formulation consisted of 7% curcuminoids (corresponding to 6% curcumin) and 93% Tween-80. Consequently a lower amount of surfactants is required in the nanoscaled Tween 80 micellar formulation than in case of the curcumin-loaded self-assembled polymeric micelles. This is of special benefit as the overall amount of surfactants administered orally may thus be reduced.

At the same time the increase in bioavailability achieved with the nanoscaled Tween 80 micellar formulation is much higher than with the curcumin-loaded-self-assembled polymeric micelles. Thus, an up to 185-fold increase in the AUC of curcumin was observed in humans with the nanoscaled micellar formulation, whereas the curcumin-loaded-self-assembled polymeric micelles achieved only a 2.87-fold increase in the AUC of curcumin compared to the native compound [10, 11, 15]. Moreover, Tween 80 is much cheaper than TPGS, rendering it a cost-effective option to enhance the delivery of poorly soluble compounds to target tissues.

## Conclusion

The present study provides insight into the processes underlying the observed 185-fold enhanced bioavailability of nanoscaled micellar curcumin formulation in two previously conducted human pharmakokinetic studies. The increase in the apparent permeability coefficient observed for micellar over native curcumin in the Caco-2 *in vitro* system results from a higher transport rate and a higher flux for micellar curcumin through the intestinal barrier. Also the amount of curcumin accumulated in the Caco-2 cells was much lower in case of nanoscaled micellar curcumin, suggesting that the micellation of curcumin does not only increase the cellular uptake but also the basolateral secretion of curcumin.
